# Editorial for the Special Issue on “Process Intensification Techniques for the Production of Nanoparticles”

**DOI:** 10.3390/nano11061534

**Published:** 2021-06-10

**Authors:** Giorgio Vilardi, Marco Stoller

**Affiliations:** Department of Chemical Engineering Materials Environment, University of Rome La Sapienza, 00184 Rome, Italy; marco.stoller@uniroma1.it

According to ISO/TS 80004, a nanomaterial is defined as the “material with any external dimension in the nanoscale or having internal structure or surface structure in the nanoscale”, with nanoscale defined as the “length range approximately from 1 nm to 100 nm”. The peculiar characteristics of nanomaterials and, in particular, of metallic and organic nanoparticles, have become of great research interest for both academic and industrial institutions. The production of nanoparticles can be performed by bottom-up or top-down processes: the former process usually starts from single atoms, ions and molecules in a fluid medium, historically belonging to wet chemistry production technologies. The latter is instead usually based on the mechanical size reduction of millimetric-size particles of the target material, by means of high-energy demanding mills. The bottom-up technologies are the most widely adopted in lab-to-pilot scale applications, whereas the top-down ones may present notable advantages for industrial scale productions. However, to achieve reproducible and homogeneous nanoparticles a number of innovative process units have been developed by various researchers, taking advantage of the particular geometry and fluid-dynamic conditions established inside the specific unit [1,2,3,4,5,6,7,8].

Most of the process units adopted for the production of nanoparticles by bottom-up technologies belong to the process intensification philosophy, since the unit is designed in order to obtain peculiar operative conditions able to maximize the mass/heat/momentum transfer inside the unit itself. Process intensification (PI) is considered as one of the most valuable development pathways for the chemical process industry, leading, generally, to an efficiency increase and plant size decrease. PI has become fundamental to reach a sustainable chemical process industry due to the increasing global demand for space, energy, health environment and more “green’’ technology. In this context, the intensification of well-known industrial activities, without increasing the land use and environmental impact, but on the contrary reducing these main drawbacks, seems to be a mandatory task for chemical engineers. Indeed, the term PI does not mean simply “increase the efficiency and reduce the plant size”, as reported above; the term itself underwent an evolution, alongside the chemical process industry, in the last two decades. In fact, PI offers several potential benefits in process improvement, the primary ones being enhancement of production efficiency and process safety considerations, lower cost, and minimization of waste at source leading to reduced environmental pollution. More in detail, one can state that PI utilizes novel chemical engineering concepts and equipment to overcome mass-transfer limitations, enhance reaction rates and therefore miniaturize process equipment. To this aim, an increased input of mechanical energy is required, in order to mix or spread the reacting fluids into thin layers with a high interfacial area in order to enhance the mass transfer rate and the consequent production rate and homogeneity. In recent years process intensification has gained importance due to its effectiveness in the utilization of resources available. Maximum conversion, high safety and minimal or optimal energy losses are the primary focus in the intensified design approach [1].

The authors that contributed to this Special Issue well report, by their work, how the process intensification philosophy can be adopted for the production of nanoparticles of great interest in various industrial and civil applications. The production of zinc oxide [3] and starch [6] nanoparticles, by means of a spinning disk reactor represent a remarkable example of intensified production in the nano-size world. The spinning disk reactor represents one of the most promising PI technologies for the heat and mass transfer rate enhancement, obtained through centrifugal fields generated in the thin liquid film on the reactor disk by the rotation action. Among the suitable SDR characteristics for nanoparticle production by precipitation methodology, the uniform and rapid micro-mixing environment generation on the rotating disk represents one of the most fundamental; the micromixing conditions provide better control on formation and local distribution of supersaturation in the liquid film, influencing nanoparticle nucleation and crystal growth kinetics (which is a function of the molecular diffusion phenomenon to the growing crystals). Another relevant production process is based on the hydrothermal route, that, according to Chen and co-workers [7] allowed the synthesis of well-dispersed zinc oxide and carbon co-modified LiFePO_4_ spherical particles (a promising cathode materials for lithium-ion batteries). The production process was demonstrated to be well reproducible, and the nanomaterials showed a notable cycling stability (initial discharge capacities of 138.7 mAh/g at 0.1 C, maintained 94.8% of the initial capacity after 50 cycles at 0.1 C).

Other technologies, green and bio-based, permit the achievement of reproducible nanomaterials, with important applications in biomedical and medical fields. In detail, Baral and co-workers [5] reported an ultra-sound assisted synthesis to produce peptide-based nanostructures [Arg-Phe]_4_ octapeptides. The nanostructures consisted of alternating arginine (Arg/R) and phenylalanine (Phe/F) sequences, that were subjected to 430 kHz ultrasound in aqueous solution in the absence of any external agents. Ibrahim and co-authors [2] instead, worked on the green synthesis of silver nanoparticles, by using the endophytic bacterium Pseudomonas poae strain CO, which was isolated from garlic plants (Allium sativum). The authors obtained nanoparticles with the size of 19.8–44.9 nm, which showed strong inhibition in the mycelium growth, spore germination, the length of the germ tubes, and the mycotoxin production of the wheat Fusarium head blight pathogen Fusarium graminearum. Podlesnaia and co-workers [8] obtained shape-anisotropic plasmonic nanoparticles, i.e., gold nano-triangles, developing a seed-mediated synthesis with high reproducibility, dramatically reducing the reaction time from 3 days down to 1 day. Finally, Puente-Massaguer and co-authors [4] demonstrated in their work that the High Five/TGE (transient gene expression) system is a suitable approach for the production of VLP (virus-like particle)-based vaccine candidates and other recombinant proteins. Initially, the authors optimized the production of HIV-1 VLPs at shake flask level, by a TGE method based on the transfection reagent polyethylenimine. Subsequently, they demonstrated the reproducibility of a TGE in High Five cell production at bioreactor scale, resulting in a higher maximum viable cell concentration (5.1 × 106 cell/mL), the same transfection efficiency and a 1.8-fold increase in Gag-eGFP VLP production compared to shake flasks.

As a conclusive work of the Special Issue, the review of Hakke and co-authors [1] summarized in an efficient and critical way the future perspectives of micro-fluidics devices in the process intensification field of nanoparticles production. The work covers the microreactors’ design as well the fundamental mechanisms involved in process intensification using these process units for synthesizing nanomaterials. The review reports a number of interesting examples, focusing on the critical evaluation of the presented results and technologies, reporting advantages and disadvantages, as well as the possible real applications up to industrial scale production of nanoparticles.

## Data Availability

Not applicable.
